# The Empirical Study of College Students’ E-Learning Effectiveness and Its Antecedents Toward the COVID-19 Epidemic Environment

**DOI:** 10.3389/fpsyg.2021.573590

**Published:** 2021-08-02

**Authors:** Cai-Yu Wang, Yuan-Yuan Zhang, Shih-Chih Chen

**Affiliations:** ^1^School of Public Health, Dalian Medical University, Dalian, China; ^2^Department of Information Management, National Kaohsiung University of Science and Technology, Kaohsiung, Taiwan

**Keywords:** higher education, e-learning strategy, e-learning effectiveness, COVID-19, structural equation model

## Abstract

Due to the impact of COVID-19, universities are forced to suspend their classes, which begin to depend on the usage of online teaching. To investigate the relationship among e-learning self-efficacy, monitoring, willpower, attitude, motivation, strategy, and the e-learning effectiveness of college students in the context of online education during the outbreak of COVID-19. A 519 first- to fifth-year undergraduate students from a medical university were selected for the research in this study. Structural equation model (SEM) was used for a data analysis, which led to the results showing that: (1) e-learning self-efficacy and monitoring have significant positive influence on e-learning strategy, and indirectly influence e-learning effectiveness through e-learning strategy; (2) e-learning willpower and attitude have a significant positive influence on e-learning motivations, and indirectly influence e-learning effectiveness through e-learning motivation and strategy; (3) e-learning motivation is having significant influence on e-learning effectiveness, while e-learning strategy is playing a mediating role; (4) There is a significant positive correlation between e-learning strategy and e-learning effectiveness; and (5) The presence of e-learning experience has a moderating influence on e-learning effectiveness as well as its influential factors. Results from this study provide the necessary information as to how higher education institutions and students can enhance students’ effectiveness of the e-learning system in order to support the usage of online technologies in the learning and teaching process. These results offer important implications for online learning effectiveness.

## Introduction

In December 2019, a kind of novel coronavirus was found in some patients with unexplained pneumonia in Wuhan, China (Li et al., 2020). The virus is highly contagious, quickly spreading all over the country, and even all over the world. On January 27th, the Ministry of Education of China also issued a notice to postpone the start of the 2020 spring semester, saying that kindergartens, primary schools, middle schools, high schools, and universities shall determine the start date of spring semester on the basis of the local situation of the epidemic control under the unified deployment of the local education authorities and government (Ministry of Education of the People’s Republic of China, 2020). Subsequently, the Ministry of Education built e-learning cloud platforms through integrating excellent educational resources across the country, and launched online teaching methods under the guidelines of the postponement of the school season without suspension of learning. Since February 17th, China’s universities have successively adopted online teaching methods to carry out teaching activities. According to USA today on March 11th, as the coronavirus outbreak was worsening, more than 100 American universities, including Harvard University, Stanford University, and Columbia University, announced the cancelation of offline courses in favor of online education. Findings from 200 countries in mid-April, 2020 showed that 94 percent of learners – 1.58 billion people – were affected by COVID-19 all over the world (United Nations, 2020). Additionally, the UNESCO (2020) reported that the closure of higher institutions has influenced over 91 percent of the students population in the world and 23.8 million students may drop out or be unable to secure admission to schools in the 2021 academic calendar. In order to alleviate the education crisis, schools around the world have adopted online teaching methods to protect the education opportunities, as well as the health and lives of students.

E-learning describes the usage of information and communication technology to develop web-based, computer, digital, or online learning (Moore, 2006; McDonald et al., 2018). In the era of the knowledge-based economy, owing to the sustainable development of information and network as well as the popularization of computers, e-learning has changed the way learners communicate, interact, and behave, and their cognition of learning (Homan and Wood, 2003). E-learning can keep working beyond the limitation of time, space, and location, which facilitates knowledge sharing between learners and teachers, thus gaining increasing numbers of applications in the field of education and having a profound impact on the development of education (Emran and Shaalan, 2014). This large-scale, open online teaching method has been developing rapidly all over the world, playing a major role in the sharing of educational resources and the promotion of educational equity (Tenório et al., 2016). During the outbreak of COVID-19, universities in China and the rest of the world adopted online teaching methods to achieve the goal of “no suspension of learning.”

Problem Statement: Online learning initiatives were a crucial step taken by many universities, provision of learning services through online technologies is now inevitable. In recent years, the research on online learning mainly includes the following three aspects: (1) the importance of online learning and the benefits it brings to students (Sheshasaayee and Bee, 2018; Panigrahi et al., 2018), (2) the acceptance of online learning, the intention of e-learning and its influencing factors (Al-Rahmi et al., 2018, 2019), and (3) the effect of online learning and its influencing factors (Gunawan et al., 2020; Nguyen et al., 2020; Pee, 2020). In terms of e-learning effectiveness, there are some attempts to improve students’ e-learning effect by improving e-learning technology, such as building Online Learning Management Systems and establishing virtual communities (Gunawan et al., 2020; Nguyen et al., 2020). Meanwhile, some works have focused on the influence of students’ characteristics and e-learning technology design (Kintu et al., 2017). These studies have confirmed the importance of e-learning in the future education development. In the same time, they play a great role in promoting the popularization of e-learning and improving students’ academic achievements through technological innovation. Different from the background of other works, since the outbreak of COVID-19, all of schools adopted the way of e-learning. There are large scale samples to investigate the effectiveness of e-learning without considering the acceptance. Therefore, to fully understand the relationships among the effectiveness of e-learning and its influence factors, in this paper, we focus on the effectiveness of college students with e-learning during the COVID-19. In previous studies, the research on e-learning effectiveness mainly focused on improving learning efficiency by updating e-learning technology, or considering students’ inherent characteristics, and seldom combined the two. There are many factors affecting the e-learning effectiveness of college students, including internal factors (i.e., learning motivations and learning strategies), and external factors (i.e., learning environment and learning monitoring) (Wang et al., 2011; Hew and Cheung, 2014). Prior works merely focus on social factors like learning environment (Bryant and Bates, 2015), or individual factors like learner’s mental factors (Lee, 2010; Lin, 2011; Huang et al., 2012; Chu and Chen, 2016). Inspired by previous studies, this paper incorporated seven influencing factors into the analysis of the effectiveness of e-learning, including the e-learning self-efficacy, e-learning monitoring, e-learning willpower, e-learning attitudes, e-learning motivations and e-learning strategies, and e-learning effectiveness. Through the questionnaire, we collect the data of college students’ e-learning attitude, self-efficacy, strategies, motivation, effectiveness and so on, and establish a structural equation model, and analyze the data through AMOS software to verify the influencing factors of college students’ online learning effectiveness.

The contributions of this paper are summarized as follows: First, in terms of research content, we consider the internal and external factors that may affect the effectiveness of e-learning, and make a detailed analysis of the internal factors of learners, which makes the research content more comprehensive. Second, in terms of research method, this study adds e-learning motivation and e-learning strategy as mediating variables to construct a more comprehensive model for analyzing the influential factors of e-learning effectiveness. Moreover, differently to other works, we propose a novel moderating variable which indicates whether you have had e-learning experience before, for further analyzing the influential factors and improving the e-learning effectiveness. This research conducts a more comprehensive analysis with these data. Last but not least, in practice, our work provides guidance for universities and students to improve the efficiency of online learning.

## Theoretical Background and Hypotheses

### E-Learning Self-Efficacy (E-LSE)

The self-efficacy theory, first proposed by the American psychologist Bandura, was defined as the evaluation of an individual’s operation ability in an activity, and that of his/her confidence and belief in whether he/she can successfully complete a task (Bandura, 1977). The concept of e-learning self-efficacy originates from computer self-efficacy and Internet self-efficacy. The advent of Internet self-efficacy, which refers to a subjective judgment of one’s ability to use the Internet, was influenced by the necessity of extending the self-efficacy from computer to Internet with the development of Internet technology (Torkzadeh and Van Dyke, 2001). Therefore, e-learning self-efficacy is a personal belief in achieving success in online learning and a kind of subjective feeling about applying computers and Internet information resources to achieve learning goals (Saadé and Kira, 2009). E-learning strategies refer to the plans for learners to consciously and purposefully adopt complex learning schemes due to the improvement of learning effects in the e-learning process (Tucker and Gentry, 2009). Studies have shown that distance learner’s learning self-efficacy has a positive predictive effect on learning strategies. Only those with high self-efficacy in e-learning can better acquire e-learning strategies and improve their online learning performance (Wang et al., 2008). The empirical research shows that there is a significant correlation between learning self-efficacy and learning strategies among junior high school students; and learning self-efficacy affects learning achievement through different learning strategies (Mahmud, 2009; Yusuf, 2011). Some studies have confirmed that great academic self-efficacy has a higher level of academic success (De la Fuente et al., 2019; Ahmadi, 2020). Therefore, we hypothesize the following:

H1: E-learning self-efficacy has a positive influence on e-learning strategy.

### E-Learning Monitoring (E-LMT)

E-learning monitoring refers to a series of processes such as inspection, evaluation, feedback, and control of students’ e-learning due to enabling learners to develop better e-learning strategies, and improve learning effects and qualities (Meyen et al., 2002). E-learning emphasizes the autonomy of learners. As external control weakens, students are very prone to spare themselves. Therefore, perfect network monitoring methods and students’ self-monitoring are particularly important. A memory-enhancing experiment on the elderly has shown that, through learning monitoring skills training, the elderly can promote the improvement of their learning strategies, and improve their learning effects by training as well (Dunlosky et al., 2003). Studies have confirmed that the utilization of self-monitoring methods by college students will affect learning effectiveness (Zhang, 2005). Therefore, we hypothesize the following:

H2: E-learning monitoring has a positive influence on E-learning strategy.

### E-Learning Willpower (E-LWP)

Learning willpower refers to the ability to overcome difficulties and to achieve one’s learning goals when encountering barriers and learning anxieties in the learning process. In the process of online education, teachers cannot immediately monitor students’ learning situation and know the degree of their knowledge mastery, so it is more necessary for students to cultivate the willpower and resist the temptation in the process of online learning, so as to achieve better learning effects. Studies have shown that adults with stronger willpower in distance learning can get better learning effects (Miller et al., 2012). An empirical study on the disabled students’ learning willpower shows that most of them hold high learning willpower, which will encourage them to obtain greater motivation and enthusiasm for learning, and is more able to resist different temptations in the learning process. The learning motivations can be enhanced by enhancing the learning willpower (Moriña et al., 2018). Therefore, we hypothesize the following:

H3: E-learning willpower has a positive influence on e-learning motivation.

### E-Learning Attitude (E-LAT)

Learning attitude refers to a kind of abstract and comprehensive mental phenomenon shown by students in the learning process, which is a persistent view with cognition, emotion, and behavioral tendency (Koballa and Crawley, 1985). The e-learning attitude hereby refers to students’ views on the e-teaching methods during the COVID-19 epidemic. Through a survey on the learning attitudes and learning motivations of high school engineering education, it was confirmed that a significant correlation between learning attitudes and learning motivations exists (Chao et al., 2015). There was a significant relationship between learning attitudes and learning effects. Students with positive attitudes toward computers acquired better learning effects than those with negative attitudes (Munger and Loyd, 1989). A study on the attitudes of eighth-grade and ninth-grade students toward learning physics and their academic achievements proved that the attitude to science is considered as an important predictor of their science achievements (Stefan and Ciomos, 2010). Therefore, we hypothesize the following:

H4: E-learning attitude has a positive influence on e-learning motivation.

### E-Learning Motivation (E-LMV)

Learning motivation refers to the motivation that will trigger and can maintain students’ learning behaviors, and enables them to complete their academic goals. It is deemed as a need to motivate and guide students to learn. E-learning motivation refers to the driving force of students in the process of online learning. There is a correlation between learning motivations and learning strategies. The students with comprehensive learning motivations are able to adopt more strategies (Sedighi and Zarafshan, 2006). A study on the relationship among learning motivation, learning strategy and academic performance of middle school students has confirmed that a significant correlation between learning motivation and learning strategy was found, and the former can indirectly affect learning performance through the latter (Megan et al., 2013). Learning motivations play a significant role in improving students’ learning effects. Studies have shown that, even with great talents, students’ poor attitudes and weak motivations will not deliver satisfactory results in language learning (Nasser and Majid, 2011). There exists a significant correlation between e-learning motivation and e-learning effectiveness; the stronger a learning motivation is, the better learning effect can take place (Özhan and Kocadere, 2020). In the study on undergraduates’ learning effects of Psychological Statistics, it has proved that there is a significant correlation among learning attitudes, motivations, and learning effects (Wang and Che, 2005). Therefore, we are going to propose the following hypotheses:

H5: E-learning motivation has a positive influence on e-learning strategy.

H6: E-learning motivation has a positive influence on e-learning effectiveness.

### E-Learning Strategy (E-LST) and E-Learning Effectiveness (E-LEC)

Empirical studies have shown that a significant positive correlation between learning strategies and learning effects does also exist. The former has a significant regressive effect and a direct impact on the latter (Lin et al., 2017; Deschênes et al., 2020). Therefore, we hypothesize the following:

H7: E-learning motivation has a positive influence on e-learning effectiveness.

E-learning effectiveness refers to the knowledge and ability acquired in the process of learning by means of network learning. Based on the exploration in the relationship between the above variables, e-learning effectiveness ought to be directly or indirectly affected by e-learning self-efficacy, e-learning monitoring, e-learning willpower, e-learning attitudes, e-learning motivations, and E-learning strategies.

### The Mediating Roles of E-Learning Motivation and E-Learning Strategy

From the above literature review on the relationships between these research variables, it can be seen that e-learning motivation and e-learning strategy can act as mediator variables through which the independent variables will influence the dependent variables. As a results, we hypothesize the following:

H8: E-learning strategy mediates the relationship between e-learning self-efficacy and e-learning effectiveness.

H9: E-learning strategy mediates the relationship between e-learning monitoring and e-learning effectiveness.

H10: E-learning motivation mediates the relationship between e-learning willpower and e-learning effectiveness.

H11: E-learning motivation mediates the relationship between e-learning attitude and e-learning effectiveness.

H12: E-learning strategy mediates the relationship between e-learning motivation and e-learning effectiveness.

H13: E-learning motivation and E-learning strategy mediate the relationship between e-learning willpower and e-learning effectiveness.

H14: E-learning motivation and e-learning strategy mediate the relationship between e-learning attitude and e-learning effectiveness.

### The Multi-Group

In the process of online learning, learners’ previous e-learning experience will influence their attitudes and outcomes. The high-quality learning outcomes obtained in previous online learning will strengthen their determinations to learn from online courses, and will help them gradually develop positive attitudes as well (Bandura, 1977). The familiarity and mastery of advanced learning methods will also influence the choice making of learning strategies. Some scholars put forward that although multimedia is not necessarily helpful for recalling knowledge, its life-oriented presentation method can lead learners to take a positive attitude with a sense of identity toward network learning, which exerts a positive impact on subsequent learning (Butler and Mautz, 1996).

**FIGURE 1:**
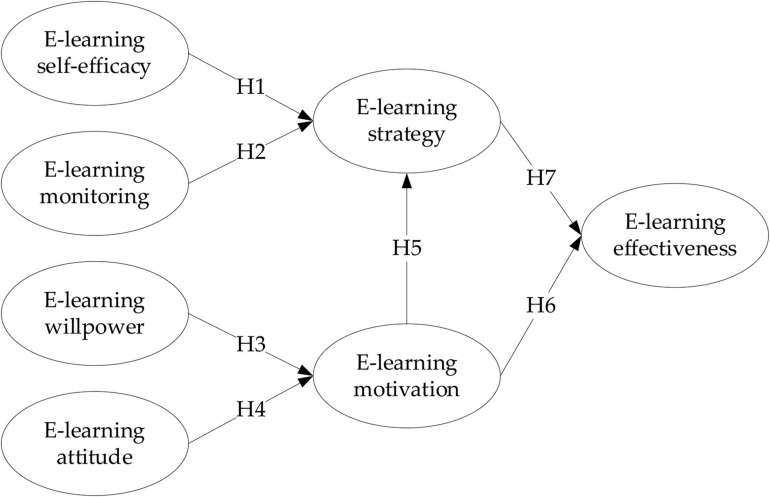
Research model.

From the above literature review and hypothesis, we have reached a complete research model, which is shown in Figure 1.

## Research Method

### Instrument

The measurement tool for e-learning self-efficacy in this study is General Self-Efficacy (GSES) (Jerusalem and Schwarzer, 1992), which has few questions and can be easily operated. According to Jerusalem and Schwarzer, with the internal consistency coefficient between 0.75 and 0.91 in multiple measurements of different cultures (countries), GSES has always kept good reliability and validity. From the GSES, we select items that can measure relevant aspects of learning and use them as the construct of e-learning self-efficacy.

The measurement tools for e-learning willpower and e-learning effectiveness are Zimmerman’s self-regulated learning theory framework (Zimmerman, 2000, 2002). The items we choose are those that can be well understood by Chinese students after translation and are in line with the characteristics of online learning.

The e-learning motivation and e-learning strategy are measured by Motivated Strategies for Learning Questionnaire (MSLQ) (Pintrich et al., 1991). The MSLQ is widely used in Chinese and foreign articles with high reliability and validity. As for the construct of e-learning motivation, we choose the items from the MSLQ scale that can reflect the intrinsic value and driving force to measure students’ learning motivation. As for the construct of e-learning strategy, we choose the items that best represent the pros and cons of the strategy, such as the formulation of a learning plan, the adjustment of the plan, the practical application of methods and the integration of learning content, etc.

The measurement tool for e-learning monitoring is borrowed from the research on the actuality of postgraduates’ independent learning on the basis of network instruction platform (Whipp and Chiarelli, 2004). The items we chose were those could be done on the existing online technology and online learning platform.

Since all colleges and universities in China have already adopted the form of online teaching because of the coronavirus epidemic, students’ attitudes toward online teaching, and whether medical students’ courses are able to well presented in the form of online courses are of great importance. So for this part, the questionnaire referred to the other scholars’ articles on students’ attitudes toward online learning (Knowles and Kerkman, 2007). The questionnaires of this paper were amended based on the literature theory and the actual situation. They are of high expert reliability with the examination and approval of several supervisors of the Department of Health Care Management of Dalian Medical University.

Likert’s seven-point scale was used in the questionnaire for self-rating, with 1–7 points indicating the degrees from “completely dissenting” to “completely consent” with a total of 31 topics included into the seven constructs.

### Sampling Procedure and Sample Structure

Considering the influence of COVID-19, this survey was carried out in the form of network questionnaire, and a stratified sampling was adopted. The questionnaires were distributed among first- to fifth-year undergraduate students from a medical university. The reason why we have chosen this university is that, it has adopted online teaching throughout the whole semester, where the students can have a complete online teaching experience, which will drive the results of this survey more authentic and reliable. Among a total of 574 finished questionnaires collected, 519 valid questionnaires were finally returned after removing those invalid questionnaires with wrong and arbitrary answers, acquiring an effective reply rate of 90.42%. Based on that, the sample size of this study (*n* = 519) is acceptable according to Hair et al. (2010), they stated the minimum sample size for quantitative research is (*n* = 300). The demographic information of respondents is shown in Table 1, that a total of 35.3% (*n* = 183) of respondents are male; while 64.7% (*n* = 336) are female. Besides, a total of 18.9% (*n* = 98) of respondents are freshman, 17.3% (*n* = 90) are sophomore, 17.9%(*n* = 93) are junior, 20%(*n* = 104) are senior, and 25.8% (*n* = 134) are fifth grade. A majority of respondents are living in urban areas (*n* = 363, 69.9%). A 30.1% respondents are living in the countryside. In terms of the device fore-learning, most of them use a phone (*n* = 275, 53%); some respondents use a computer (*n* = 129, 24.9%), and others use an Ipad (*n* = 115, 22.2%). Most respondents have e-learning experience (*n* = 344, 66.3%); while 175 respondents have no E-learning experience (33.7%).

**TABLE 1:** Demographic characteristics of respondents (*n* = 519).

**Variables**	**Category**	**Frequency**	**Percentage**
Gender	Male	183	35.3
	Female	336	64.7
Grade	Freshman	98	18.9
	Sophomore	90	17.3
	Junior	93	17.9
	Senior	104	20.0
	Fifth year	134	25.8
Living area	City	363	69.9
	Countryside	156.0	30.1
E-learning equipment	Phone	275	53.0
	Computer	129	24.9
	Ipad	115	22.2
E-learning experience	Yes	344	66.3
	No	175	33.7

*Ps: China’s undergraduate medical major is a 5-year system.*

## Results

In order to ensure the reliability of questionnaires, the valid part have been coded and registered, and were analyzed by using SPSS25.0. Meanwhile, AMOS24.0 was used to establish the structural equation model and analyze the data, thus discussing the causal relationship among e-learning self-efficacy, e-learning monitoring, e-learning willpower, e-learning attitude, e-learning motivation, e-learning strategy and e-learning effectiveness; and the fitting degree of the model was tested on the basis of path analysis. Finally, the structural equation model analyzes whether learners’ previous e-learning experience will influence their attitudes and outcomes.

### Reliability and Validity Analysis

Structural equation modeling (SEM) provides a maximum-likelihood estimation of the entire system in a hypothesized model, and enables the assessment of variables with the data. First, the measurement model was confirmed by using confirmatory factor analysis (CFA); and then we performed SEM analysis to measure the fit and path coefficients of the hypothesized model. Based on the Suggestions of Jöreskog and Sörbom (1989), the items with factor loading less than 0.6 were deleted (Hair et al., 2017). As a result, E-LSE5, E-LWP5, E-LST5, E-LEC4, and E-LEC5 were deleted. After the amendments, all constructs in this model could satisfy the requirements for reliability. The questionnaire is shown in Appendix. The results of analysis show that the factor loading of all the dimensions is ranged between 0.676 and 0.938, which is very significant and meets the requirements.

We will keep each item for internal consistency analysis; and Cronbach’s alpha values are ranged between 0.812 and 0.926, higher than 0.7 (Nunnally and Bernstein, 1994). Composite reliability (CR) is ranged between 0.817 and 0.928, which is higher than 0.7 (Werts et al., 1974; Gefen et al., 2000; Kline, 2010). Average variance extracted (AVE) is between 0.611 and 0.764, higher than 0.5 (Hair, 2010). The reliability and validity of the model is good; and the specific values are shown in Table 2.

**TABLE 2:** Convergent validity of the measurement model.

**Construct**	**Item**	**Mean**	**S.D.**	**Factor loading**	**Cronbach’s alpha**	**Composite reliability**	**Average variance extracted (AVE)**
E-learning self-efficacy (E-LSE)	E-LSE1	5.471	1.383	0.784	0.899	0.900	0.694
	E-LSE2	5.298	1.412	0.880			
	E-LSE3	5.312	1.380	0.890			
	E-LSE4	5.451	1.243	0.771			
E-learning monitoring (E-LMT)	E-LMT1	5.127	1.271	0.813	0.812	0.817	0.611
	E-LMT2	5.344	1.17	0.845			
	E-LMT3	4.976	1.458	0.676			
E-learning willpower (E-LWP)	E-LWP1	4.742	1.389	0.898	0.917	0.919	0.740
	E-LWP2	4.681	1.451	0.884			
	E-LWP3	5.013	1.262	0.850			
	E-LWP4	4.758	1.414	0.805			
E-learning attitude (E-LAT)	E-LAT1	4.962	1.519	0.938	0.885	0.896	0.731
	E-LAT2	4.653	1.607	0.909			
	E-LAT3	5.167	1.335	0.697			
E-learning motivation (E-LMV)	E-LMV1	5.763	1.068	0.857	0.865	0.864	0.616
	E-LMV2	5.549	1.115	0.841			
	E-LMV3	5.641	1.081	0.695			
	E-LMV4	5.743	1.095	0.735			
E- -learning strategy (E-LST)	E-LST1	5.202	1.179	0.860	0.904	0.911	0.721
	E-LST2	5.067	1.175	0.894			
	E-LST3	4.965	1.216	0.892			
	E-LST4	4.764	1.431	0.741			
E-learning effectiveness (E-LEC)	E-LEC1	5.152	1.188	0.913	0.926	0.928	0.764
	E-LEC2	5.263	1.151	0.920			
	E-LEC3	5.068	1.232	0.847			
	E-LEC4	5.382	1.265	0.812			

### Discriminant Validity

According to the suggestions by some scholars such as Fornell and Larcker (1981) and Hair (2010), the criterion for deciding whether each construct has discriminant validity is to see if the square root of the average variance extracted (AVE) of the construct can be greater than the correlation coefficient between other constructs. As shown in Table 3, the diagonal boldface represents the square root of the AVE value of each construct. These values are greater than or close to the correlations of other constructs. Therefore, the psychometric characteristics of the instrument are acceptable in terms of discriminant validity.

**TABLE 3:** Discriminant validity.

**Construct**	**AVE**	**E-LSC**	**E-LMV**	**E-LST**	**E-LAT**	**E-LWP**	**E-LMT**	**E-LSE**
E-LSC	0.764	**0.874**						
E-LMV	0.616	0.680	**0.785**					
E-LST	0.721	0.933	0.673	**0.849**				
E-LAT	0.731	0.724	0.555	0.667	**0.855**			
E-LWP	0.740	0.702	0.660	0.717	0.685	**0.860**		
E-LMT	0.611	0.783	0.615	0.814	0.565	0.650	**0.781**	
E-LSE	0.694	0.660	0.594	0.636	0.494	0.572	0.578	**0.833**

*The shaded numbers in the diagonal row are square roots of the AVE. Off-diagonal elements are the correlations among constructs.*

### Assessment of the Structural Model

The model fitting degree index is mainly used to analyze the degree of fitting between the theoretical model and the sample data. The smaller the chi-square value is, the better, but there is no certain standard because the chi-square value will be affected not only by the number of samples, but also by the complexity of the model. Therefore, the chi-square value in this paper is deemed as acceptable (χ2 = 1096.48). The more degree of freedom, the better (df = 286). In this model, the value of χ2/df is 3.834, which is less than 5, which is acceptable. Both CFI (0.930) and TLI (0.920) values are greater than 0.9, which is acceptable. The GFI (0.852) value is close to 0.9, which is barely acceptable. RMSEA value is 0.074, less than 0.08, which is accepted. The model fit is adequate for the empirical data.

**FIGURE 2:**
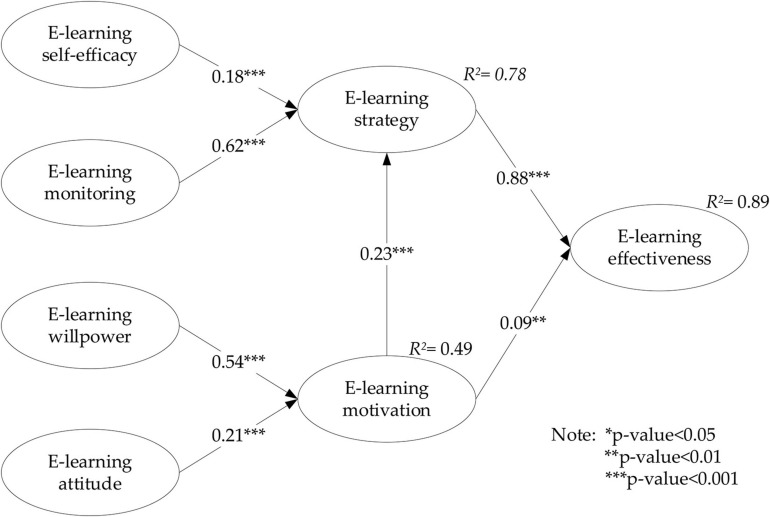
Structural model result.

The structural model assessment as shown in Figure 2 and Table 4 provides the indication of the hypothesis tests. E-learning self-efficiency significantly predicts e-learning strategy. Hence, H1 is accepted with (β = 0.177, *p* < 0.001). Likewise, e-learning monitoring significantly predicts e-learning strategy. Hence, H2 is supported (β = 0.625, *p* < 0.001). These are quite similar with e-learning willpower and e-learning attitude which have been found to significantly influence e-learning motivation. Hence, H3 and H4 are accepted with (β = 0.543, *p* < 0.001) and (β = 0.206, *p* < 0.001), respectively. E-learning motivation significantly predicts e-learning strategy. Hence, H5 is supported (β = 0.225, *p* < 0.001). E-learning motivation significantly predicts e-learning effectiveness. Hence, H6 is supported (β = 0.09, *p* < 0.005). E-learning strategy significantly predicts e-learning effectiveness. Hence, H7 is supported (β = 0.883, *p* < 0.001). As a result, H1, H2, H3, H4, H5, H6, and H7 are supported. Among all the hypotheses, the e-learning strategy has the greatest influence on the e-learning effectiveness.

**TABLE 4:** Structural path analysis result.

**Hypothesis**	**Relationship**	**Path coefficient**	**Estimate**	**S.E.**	**C.R.**	**P**	**Hypothesis testing result**
H1	E-LSE→ E-LST	0.177	0.171	0.037	4.582	***	Supported
H2	E-LMT → E-LST	0.625	0.626	0.049	12.808	***	Supported
H3	E-LWP → E-LMV	0.543	0.392	0.042	9.381	***	Supported
H4	E-LAT → E-LMV	0.206	0.127	0.034	3.748	***	Supported
H5	E-LMV → E-LST	0.225	0.264	0.043	6.101	***	Supported
H6	E-LMV → E-LEC	0.09	0.109	0.039	2.823	0.005	Supported
H7	E-LST → E-LEC	0.883	0.909	0.039	23.261	***	Supported

***p < 0.001. E-LSE, E-learning self-efficiency; E-LST, E-learning strategy; E-LMT, E-learning monitoring; E-LWP, E-learning willpower; E-LAT, E-learning attitude; E-LMV, E-learning motivation; E-LEC, E-learning effectiveness.

### Mediation Effect Analysis

Regarding the mediation hypotheses (indirect hypotheses), among the variety of testing methods, the most widely used method shall be the causal step approach popularized by Baron and Kenny (1986). They mentioned that a variable will function as a mediator when it meets the following conditions: (1) the predictor variable must significantly predict the outcome variable when the mediator is excluded; (2) the predictor variable must significantly predict the mediator; (3) the mediator must significantly predict the outcome variable; and (4) the predictor variable must predict the outcome variable less strongly when the mediator is entering the model. However, many problems still exist. Most notably, simulation studies have shown that among the methods for testing intervening variable effects, the causal steps approach is among the lowest in power (Fritz and MacKinnon, 2007). The other approach is the Sobel test, in spite of a major drawback in this test. It requires the assumption that the sampling distribution of the indirect effect is normal, but the sampling distribution of the surface is often asymmetric, with non-zero skewness and kurtosis (Sobel, 1982, 1986; Bollen and Stine, 1990; Stone and Sobel, 1990). Simulation research shows that the bootstrapping method tends to own the highest power and the best Type I error control, and is already implemented in some SEM software like AMOS. Therefore, we shall focus on bootstrapping as the best option (Lockwood and MacKinnon, 1998; MacKinnon, 2000, 2012).

Table 5 shows the result of the bootstrapping analysis, indicating that the total effect point estimation (β) = 1.145 was significant with a Z of 14.870. Preacher and Hayes indicated that when the 1.145, 95% Boot CI: bias-corrected (LL = 1.001, UL = 1.309), percentile (LL = 1.000, UL = 1.304) do not straddle a 0 in between, which indicates that there is a mediation. In the model of e-learning self -efficacy affecting E-learning effectiveness through e-learning strategy, β = 0.155, *Z* = 2.981 > 1.96, 95% Boot CI do not straddle a 0 in between. Thus, this study can be concluded that the mediation effect of e-learning strategy is statistically significant between e-learning self-efficacy and e-learning effectiveness, indicating that H8 is supported. The results of H9 reveal that the mediation effect of e-learning strategy is statistically significant between e-learning monitoring and e-learning effectiveness (β = 0.569, *Z* = 8.014, 95% Boot CI do not straddle a 0 in between), so H9 is supported. A test of H10 and H11 proves that the mediation effect is not significant with β = 0.043, *Z* = 1.955, 95% Boot CI do straddle a 0 in between and β = 0.014, *Z* = 1.400, 95% Boot CI do straddle a 0 in between, respectively, so H10 and H11 is not supported. The results of H12 reveal that the mediation effect of e-learning strategy is statistically significant between e-learning motivation and e-learning effectiveness (β = 0.240, *Z* = 3.692, 95% Boot CI do not straddle a 0 in between), so H12 is supported. A test of H13 proves that the mediation effect is significant (β = 0.094, *Z* = 3.357, 95% Boot CI do not straddle a 0 in between), indicating that the mediation effect of e-learning motivation and e-learning strategy is statistically significant between e-learning willpower and e-learning effectiveness, so H13 is supported. The results of H14 reveal that the mediation effect of e-learning motivation and e-learning strategy is statistically significant between e-learning attitude and e-learning effectiveness (β = 0.030, *Z* = 2.000, 95% Boot CI do not straddle a 0 in between), so H14 is supported.

**TABLE 5:** Standardized indirect, and total effects of the hypothesized model.

**Hypothesis**	**SIE**	**Point estimation**	**Product of coef.**		**Bootstrap 5000 times 95% CI**			
					**Bias-corrected**		**Percentile**	
			**SE**	**Z**	**Lower**	**Upper**	**Lower**	**Upper**
H8	E-LSE→E-LST→E-LEC	0.155	0.052	2.981	0.062	0.266	0.056	0.259
H9	E-LMT→E-LST→E-LEC	0.569	0.071	8.014	0.439	0.714	0.445	0.725
H10	E-LWP→E-LMV→E-LEC	0.043	0.022	1.955	0.004	0.091	−0.002	0.084
H11	E-LAT→E-LMV→E-LEC	0.014	0.010	1.400	0.001	0.043	0.000	0.038
H12	E-LMV→E-LST→E-LEC	0.240	0.065	3.692	0.123	0.377	0.118	0.372
H13	E-LWP→E-LMV→E-LST→E-LEC	0.094	0.028	3.357	0.048	0.160	0.044	0.153
H14	E-LAT→E-LMV→E-LST→E-LEC	0.030	0.015	2.000	0.008	0.068	0.006	0.062

### Multi-Group Analysis

In this paper, the overall sample is divided into two parts based on the moderating variable of e-learning experience. The group 1 stands for the students with e-learning experience, while the group 2 stands for the students without e-learning experience. Then, we are going to analyze the e-learning effectiveness and its influencing factors by testing whether the factor loading, variances and residuals of the two groups are equal, that is, whether the e-learning experiences have moderating influence on the e-learning effectiveness and its influencing factors.

In factorial invariance analysis, a baseline model needs to be established prior to any invariance constraints. If the baseline model of each group is different, then the factorial invariance analysis procedures must not be conducted. On the other hand, if the baseline model is the same for each group and cannot be rejected in each group, the restrictive constraints can then be imposed on the model. First, factor loadings were constrained to be equal across the groups to test for invariance of the factor loadings. If the factor loading constrained model was acceptable, then unique variances of each item would be constrained to be equal across the groups. Finally, if factor loadings and unique variances of each item were equal across both groups, factor variance would be constrained to be equal across gender.

As shown in Table 6, since the two baselines model for each group were the same, multi-group analysis was then conducted. Firstly, a multi-group analysis with the unconstrained model showed an acceptable baseline model for the two groups (χ*2* = 1618.188, df = 579, TLI = 0.899, CFI = 0.91, RMSEA = 0.059, *p* < 0.05). Then, in order to test the invariances of the factor loadings across the two groups, factor loadings were constrained to be equal across the two groups. The χ*2* difference test between baseline model and constrained model was significant (Δχ*2* = 39.482, Δdf = 19, *p* < 0.05), which suggested that factor loadings of both groups should be variant.

**TABLE 6:** Invariance analysis of E-learning effectiveness across experience.

**Two groups**	**With E-learning experience. Without E-learning experience**	**χ*2***	**df**	**TLI**	**CFI**	**RMSEA**	**Nested models**	**Δχ*2***	**Δdf**	**Significance level**
1	Unconstrained	1618.188	579	0.899	0.91	0.059				
2	Factor Loading invariance	1657.67	598	0.9	0.908	0.059	2–1^a^	39.482	19	0.004
3	Factor Loading invariance and unique variance	1758.704	624	0.898	0.902	0.059	3–2	101.034	26	0.000
4	Factor Loading invariance and unique variance and factor variance	1782.282	634	0.898	0.901	0.059	4–3	23.578	10	0.009

*2–1^*a*^ refers to Model 2 (factor loading invariance) being more restrictive or nested within Model 1 (Unconstrained).*

In addition to the factor loadings, the unique variances of each item were constrained to be equal across the two groups as well. The χ*2* difference test between the two constrained models was significant (Δχ*2* = 101.031, Δdf = 26, *p* < 0.05). This suggested that, aside from the factor loadings, unique variances of each item should also be variant across experience.

Finally, besides the above constraints mentioned, factor variances were also constrained to be equal across the two groups. The χ*2* difference test between the two constrained models was significant (Δχ*2* = 23.578, Δdf = 10, *p* < 0.05). Therefore, all these results have revealed that the factor loadings, unique variances and factor variances were variant across two groups. That means the moderating role of the e-learning experience exists. So, the e-learning experience has moderating influence on the e-learning effectiveness, together with its influential factors.

## Discussion and Conclusion

### Discussion

The study results have shown that college students’ e-learning self-efficacy has a significant positive influence on e-learning strategies, and provides with the indirect influence on e-learning effectiveness through e-learning strategies, which is consistent with the conclusions of relevant studies (Wang et al., 2008). This may be owing to the students with higher sense of self-efficacy, who are more confident in themselves and used to adopt positive and comprehensive learning strategies for improving their learning effectiveness. Therefore, we shall pay close attention to cultivating college students’ e-learning self-efficacy. The e-learning self-efficacy can affect subsequent behaviors, but it is affected by the results of the previous behaviors as well. A long-lasting period of negative learning results will thwart learners’ self-efficacy. As Bandura stressed, self-efficacy is not an individual’s assessment of what skills or abilities one has, but a judgment of one’s confidence in what kind of skills or abilities used to complete a specific task. In this regard, schools and teachers should help students build more confidence by reasonably arranging learning content of different difficulty levels, from easy to difficult, step by step. And, a series of incentive measures, such as goal incentive, affective encouragement, and competition-cooperation incentive are encouraged to be adopted for the purpose of providing learners with successful experience and enhancing their confidence.

The transformation of learning concepts and methods has also changed the original places of teaching and learning, endowed with more emphasis on “learning” over “teaching.” In view to this, a learning-oriented teaching model should be adopted. Attention should be paid not only to learners’ learning effectiveness, but also to learners’ internal cognition and emotion. Therefore, instead of only focusing on academic performances, we should also build up a diversified teaching valuation system to tap into and develop students’ potentials in various aspects, thus helping students identify themselves and enhance their self-confidence, so as to achieve the multi-dimensional and multi-level training objectives in terms of “cognition, emotion and skill” (Kiliç-Çakmak, 2010).

From the above analysis results, this study found that e-learning monitoring has a significant influence on e-learning strategies, and offers indirect influence on e-learning effectiveness through e-learning strategies, which is consistent with the conclusions of relevant studies (Zheng et al., 2018). In addition, among all influential factors, the most influential factor is e-learning monitoring. In a traditional teaching model, the monitoring on students’ learning state comes from teachers, which is a face-to-face, real-time monitoring with good effects. Amid the COVID-19 epidemic, however, for the sake of the students and teachers’ life and health, the adoption of network teaching model separates them apart from each other and keeps the students in a virtual teaching environment, which makes it harder for students to learn and communicate with each other. Moreover, students’ unfamiliarity with e-learning technology might easily lead to reduced learning interest and academic lassitude, which is not conducive to the development of effective e-learning strategies and has an impact on e-learning effectiveness. Therefore, only by strengthening e-learning monitoring can we effectively guarantee the formulation of learning strategies, and achieve higher learning effectiveness (May et al., 2011; Rafart et al., 2019).

In order to strengthen the e-learning monitoring, works can be done from two aspects. On the one hand, the external monitoring could place constraints on learners. The e-learning platform used by students should not only monitor the learning time, login time, course-viewing progress, homework submission, classroom interaction, and so on, but also provide learning records of other students in the whole class or in the whole school, so that learners can take it as a reference to timely understand their own learning situation, and to adjust their learning strategies. Teachers, as the core part of the teaching process, should improve their participation during online education, answer questions in time, organize forums frequently, communicate and discuss with students on certain issues, and have a good understanding of students’ learning state (Lee et al., 2012). On the other hand, learners should strengthen self-monitoring – a spontaneous cognitive feature. E-learning self-monitoring requires the inspiration and intervention of students to improve their self-consciousness. Students are encouraged to check themselves, and write a self-examination diary every day to reflect on their learning state, so as to achieve the effects of self-monitoring (Metz et al., 2012).

E-learning willpower has a significant positive impact on e-learning motivations, and e-learning effectiveness is positively affected by e-learning willpower through e-learning motivations and e-learning strategies. The learning behaviors in university mainly depends on students’ autonomous learning ability. During the epidemic period, the adoption of online teaching method makes the learning willpower especially important. The lack of willpower makes it difficult to overcome the temptation in the process of online learning. Without a clear goal to strive for, it will lead to insufficient learning motivations, inefficient learning strategies, and ultimately poor learning effectiveness. Therefore, it is necessary for students to cultivate e-learning willpower and develop good learning habits. The habit is a huge force that can dominate life. The development of good habits can help shape an intense e-learning willpower (Fitch and Ravlin, 2005). So it will help one be adapted to online education better to master e-learning methods, get familiar with network technology and develop suitable learning methods for oneself.

The study results show that e-learning attitude has a significant positive impact on e-learning motivations, and e-learning effectiveness is positively affected by e-learning attitude through e-learning motivations and e-learning strategies. Which is consistent with the conclusions of relevant studies (Sridharan et al., 2010; Tarhini et al., 2014). At present, great progress has been made in the infrastructure construction and resource development of educational informatization, which makes distance education develop rapidly in the world and become a mainstream trend. During the outbreak of COVID-19, online teaching is the only choice, and after the outbreak, it will be an important supplement to offline teaching. Therefore, we should attach great importance to e-learning, with a positive and serious attitude toward every e-learning course, and achieve remarkable results.

### Conclusion

From the above analysis, it can be shown that e-learning motivations significantly positively affect e-learning effectiveness, together with e-learning strategies playing a mediating role among them. Students with strong e-learning motivations are inclined to adopt comprehensive and efficient e-learning strategies, and their e-learning effectiveness is also higher. For the purpose of improving college students’ e-learning motivation, it is necessary to activate their interests in learning since interest is the best driving force that guides them to gain some exploratory and active learning strategies, and also use these methods actively and creatively in the process of online learning. Meanwhile, in the process of online teaching, teachers can make the classroom lively and interesting by enhancing interaction and organizing games. They should also know what kind of learning content students are interested in. Students should also actively think about and set learning objectives for themselves. What is more, they should take practical actions to achieve them (de Leeuw et al., 2019).

The study results show that e-learning strategies have a direct positive impact on e-learning effectiveness. Given this, college students should adopt efficient and comprehensive e-learning strategies in the process of online learning. Before online learning, they should have a general understanding of what will be learned and make a learning plan accordingly. During the learning process, they should adjust the plan timely when they find it not in harmony with reality (Erenler, 2020). Afterward, they shall classify and summarize what they have learned, actively communicate with classmates, and share e-learning experience so as to learn from each other (Fee, 2013).

E-learning experience is a moderator variable on learning effectiveness as well as its influential factors. The two groups, with or without e-learning experience, vary a lot in learning effectiveness and its influential factors, which therefore shows the importance for students to gain more e-learning experience. Therefore, in the face of the developing trend of the times, we should keep enriching our e-learning experience. Students who have no e-learning experience should be proficient in using the online learning platform before online teaching, and understand how to solve technical problems in the online learning platform. In addition, ask experienced students what materials or skills they need to prepare in advance, and finally increase the frequency of e-learning, participating in more formal or informal online teaching tasks, and enriching the learning experience (So et al., 2019). Students with e-learning experience need to improve the depth and efficiency of online learning, and achieve their learning goals by cultivating appropriate learning strategies.

### Limitations and Future Directions

This study has several limitations that leave open future research directions. First of all, this study used cross-sectional data to examine the theoretical model and all data were collected from one source. Although the statistical analysis results suggest that common method bias may not be a concern in this study, future studies could take a longitudinal approach and collect data in different periods from different sources, in order to further confirm the causal relationship among the constructs. Second, the efficiency of online learning may also be affected by other factors like the objective environment, emotions and so on, so more variables ought to be included. Last but not least, medicine is an important means to ensure humans’ health and life safety, therefore among them, medical students are playing a vital role. Medicine in the twenty-first century was expected to “hit the ground running,” so the training process of medical students not only required traditional clinical education, but also one that was up-to-date with the latest technologies in order to ensure flexibility in a dynamic workplace. Therefore, we have chosen medical students as the survey subjects. However, in future research, more students in different disciplines should be investigated to make the research more widely applicable. Finally, considering this study has raised many interesting questions, it is believed that the current study triggers additional theorizing and empirical investigation on e-learning effectiveness, as well as its influential factors.

## Data Availability Statement

The raw data supporting the conclusions of this article will be made available by the authors, without undue reservation.

## Ethics Statement

Ethical review and approval was not required for the study on human participants in accordance with the local legislation and institutional requirements. The patients/participants provided their written informed consent to participate in this study. Written informed consent was obtained from the individual(s) for the publication of any potentially identifiable images or data included in this article.

## Author Contributions

C-YW and Y-YZ contributed to research design, performed the sample collection, data analysis, and conducted the research design. C-YW, Y-YZ, and S-CC wrote the manuscript. All authors read and approved the final manuscript.

## Conflict of Interest

The authors declare that the research was conducted in the absence of any commercial or financial relationships that could be construed as a potential conflict of interest.

## Publisher’s Note

All claims expressed in this article are solely those of the authors and do not necessarily represent those of their affiliated organizations, or those of the publisher, the editors and the reviewers. Any product that may be evaluated in this article, or claim that may be made by its manufacturer, is not guaranteed or endorsed by the publisher.

## Appendix

Instrument for variables

**Table:**

**Construct**	**Measurement items**	**Sources**
	E-LSE1: If I try my best, I can always solve the problem	
E- -learning self-efficacy	E-LSE2: I can calmly face difficulties, because I believe in my ability to deal with problems	CSES Jerusalem and Schwarzer, 1992
(E-LSE)	E-LSE3: For me, sticking to ideals and achieving goals is a breeze	
	E-LSE4: Compared with other students in the class, I hope my academic performance is better	
E- -learning monitoring (E-LMT)	E-LMT1: E-learning platform has good monitoring of learning time, progress, etc.	Whipp and Chiarelli, 2004
	E-LMT2: Teachers have good monitoring of your learning	
	E-LMT3: You often reflect on deficiencies in the learning process and correct	
E- -learning willpower (E-LWP)	E-LWP1: When I study online, I can devote myself to learning without thinking about anything else	Zimmerman, 2000, 2002
	E-LWP2: When learning online, I will not be attracted by entertainment information	
	E-LWP3: I can overcome the troubles in E-learning and adjust my emotions to continue learning	
	E-LWP4: When studying online, my friends came to chat with me, but I still insist on learning	
E-learning attitude (E-LAT)	E-LAT1: I like the new form of online teaching	Knowles and Kerkman, 2007
	E-LAT2: I can adapt well to online teaching methods	
	E-LAT3: The content of the textbook can be better presented in the form of network	
E- -learning motivation (E-LMV)	E-LMV1: I think what I have learned in the classroom is helpful for the growth of knowledge	MSLQ Pintrich et al., 1991
	E-LMV2: I am interested in what I have learned	
	E-LMV3: What I have learned is very important for the final exam	
	E-LMV4: What I have learned is very important for my future work	
E- -learning strategy (E-LST)	E-LST1: Before E-learning, I will make a learning plan	MSLQ Pintrich et al., 1991
	E-LST2:When I find that the plan is not in harmony with reality, I will immediately adjust the learning plan	
	E-LST3:I will apply the successful methods in the past homework and real class to E-learning	
	E-LST4:When learning new content, I tried to combine other content	
E- -learning effectiveness (E-LEC)	E-LEC1: I have a good grasp of the knowledge on the online course	Zimmerman, 2000, 2002
	E-LEC2: I fully understand what I have learned	
	E-LEC3: I will be able to cope well with the exam	
	E-LEC6: E-learning enriches my learning style	
